# Life as an early career researcher: interview with Kavita Beri

**DOI:** 10.4155/fsoa-2016-0012

**Published:** 2016-03-15

**Authors:** Kavita Beri

**Affiliations:** 1 BE Skin & Laser Medspa, Ocean, NJ 07712, USA; 2 Center for Dermal Research; Department of Biomaterials, Rutgers: The State University of New Jersey, 795 Bevier Road, Piscataway, NJ 08854, USA; 3 Department of Medicine, Jersey Shore University Medical Center, 1945 art 33 Neptune, NJ 07753, USA

**Keywords:** career, interview, Physician scientist, regenerative medicine, Researcher, stem cells, research

## Abstract

**Kavita Beri speaks to Francesca Lake, Managing Editor.** Kavita Beri is a board certified Internal Medicine physician in the United States, and is an active member of the American Society of Laser Medicine and Surgery. She is the medical director and owner of BE Skin & Laser Med Spa, an aesthetic medicine practice in NJ, USA. She holds a position as a Visiting Scientist at the Department of Biomaterials and Center for Dermal Research at Rutgers University (NJ, USA) and is an active staff member in the Department of Medicine at Jersey Shore University Medical Center (NJ, USA). She holds two research patents and has presented her research at international regenerative medicine conferences. She is a frequent contributor to biomedical literature, with particular research interests in tissue engineering with stem cells combined with lasers, including cancer stem cells, and medicinal properties of plant stem cells in wound healing and skin regeneration. She is an avid practitioner of Yoga and holds a strong interest in how Yogic and Vedic philosophy may guide scientific intuition and discovery.

## Q Can you tell us about your career path to date?

I chose to do medicine very early in my career as it was a science field that had so much interaction with life and people. Finishing medical school, residency and then owning my own business of an aesthetic practice has been a very fulfilling journey. Research in science has always been my driving force, my connection with the ‘realm of the unseen’. It helps dissociate from a more physical visual perspective to a more hypothetical and theoretical aspect of science.

## Q What did you find most challenging when establishing yourself in medicine?

I think the most challenging aspect is coming out of medical school/residency and creating a niche for yourself in your practice. Discovering your true passion and just following it is key. Becoming an entrepreneur not only needs some business sense but also social skills, and the ability to influence and to create a brand – these are not things you learn in medical school. My practice BE Skin & Laser Medspa (NJ, USA) represents every aspect of my vision of where skincare and antiaging cosmetics should be focused in the future.

## Q Can you tell us more about what your current position entails, & what you are working on?

My current research is based on skin/wound healing with use of laser and certain plant stem cell-derived molecules.

I have two patents. One is called Neo Niche™ (Beri Esthetique LLC, NJ, USA) which is a method to increase dermal collagen combining laser heat with a plant stem cell extract and *N*-acetylglucosamine. The second patent is a drug, BE 818, which has a compound derived from plants that we are testing to help in tissue regeneration. The two patents are designed to be used in biomaterial and tissue regeneration research and eventually in cosmetic skin care and scar healing. My goal is to use the drug and the laser heat to affect the environment around stem cells in the epidermal and dermal layers in order to regenerate into fibroblast cells and help in collagen synthesis/scar correction and wound healing. I envision that the drug will be used as coating in conjunction with skin grafts and biomaterials; its application will be both *in vivo* and *in vitro* organogenesis. I believe through nature; and following nature's laws, we will be able to progress the science of regenerative medicine beyond its years. Plants and their inherent regenerative capacity have a lot to offer our world, and we are only slowly grasping nature's full potential. Just as nature is ever birthing and ever providing, we have to see beyond the obvious and appreciate its uniqueness.

## Q How did you come to set up your clinical practice?

My passion, as I discovered while in training, was to focus on aging physiology and regeneration. With the skin being such a dynamic part of the body, it amazed me how its functioning was influenced by aging, and the entire physiology of normal aging. I enjoyed learning, performing aesthetic laser procedures and healing skin both aging skin and scars – a big aspect of antiaging medicine.

I wanted to provide services that incorporated not only maintaining healthy skin but also healthy aging. The practice represents the importance of healthy skin but with a more holistic and natural approach incorporating products/laser procedures and other aesthetics services that have a strong scientific backing. I also plan on incorporating yoga as a part of the services we can provide to our patient population, so that we could cover the whole holistic approach to antiaging. I wanted my practice to represent both science and clinical aspect of medicine along with the aesthetic appeal. It allows me to have a balance in both aspects of my career – research and clinical medicine.

## Q How do you find your work in the clinic affects the way you do research, & vice versa?

My clinical work is where I would expect to see the clinical application of the theories we test in research. In a way, one is dependent on the other and always influences and drives the other forward. In my case, I think my strongest suite is in the fact that I am able to do both at the same time and it is a very rewarding experience.

Being a clinician and a researcher, I definitely think both complement each other. Keeping an open mind, and reasoning in all aspects of your clinical practice can influence new ideas to the more creative and researcher aspect of me. I feel I learn something from each patient that walks through my office, and each case I do. The researcher in me likes to be able to ‘see beyond the seen’. That is where I believe new ideas originate. So I would not do one without the other!

## Q Where do you see yourself in 10 years?

I had always envisioned myself to be able to contribute new science to the regenerative medicine field – become an expert on stem cells. I do envision myself as always being the scientist/researcher – always learning and exploring the beauty that is life.

## Q What do you plan on doing in order to achieve your status as a stem cell expert?

The patents I have are meant to influence current research in stem cell science of tissue regeneration, wound healing and scar management. I am actively involved, and of course always open to new collaborations in the stem cell field to test the ideas and create new science. Keeping myself updated with current research as well as working with biomaterial companies on new products and devices is definitely key.

## Q What do you find most rewarding about your work?

The most rewarding aspect of my work clinically is obviously the patients I interact with. With my research work, the most enjoying aspect of the work is the ability to be able to have autonomy on my research, to be able to design a study and bring together a thesis to prove. I enjoy reading journals and exploring new ideas and I believe we influence each other with new ideas and possibilities when we are open to new information.

## Q What would you say are the biggest challenges facing early-career researchers, from your point of view?

I really believe that the biggest challenge is establishing a platform once you have an idea base. Funding and support from industry are very important and definitely challenging. However, I do believe it is all in the attitude, and as long as you have passion, you will make it.

Pitching an idea to a funding source is an art; you learn over time. Larger scale research requires funding, and with a lot of the industry in biomaterials and wound healing being startups, there is a competitive market within which a researcher needs to gain support for their ideas. I would applaud platforms where a clinician–researcher could pitch ideas to biomaterial companies working with stem cells, and thus facilitate collaborations.

## Q What advice would you give to early-career researchers looking to establish themselves in their career?

I would say, moments of inspiration beckon creativity within us. If you are inspired, use that drive to dwell within and derive ideas from your substance, your core and your passion. Not giving up on your vision, believing in yourself and respecting yourself are stepping stones to moving forward. Perseverance and a positive attitude go a long way. Just from experience, I would say that we should accept every opportunity as just that, and not a coincidence, as we can trust the universe to show us our path. Looking for avenues to market your expertise and honing social skills regardless of your field mark successful traits, even in laboratory-based researchers. Having a voice and using social media tools to help network with important leaders in your field of interest are some new ways to explore horizons.

## Q There is often media surrounding gender imbalances in science. Do you see this, & if so has it affected you at all?

I personally believe that a mind is what creates an idea and that has no gender. As long as we can look beyond the physical and see the substance that has created a vision/idea in someone's mind, the gender bias issue would soon be insignificant. In reality, as a female we are challenged to juggle many aspects/responsibilities with family and career. I do applaud women who create the path they wish to follow despite all odds, and many in the field of science are women that have influenced me and my career choices.

## Q Finally, if you could go back in time, what advice would you give your younger-self in terms of your career?

Read, read, read and read more…indexing services such as PubMed have it all!

